# HGprt deficiency disrupts dopaminergic circuit development in a genetic mouse model of Lesch–Nyhan disease

**DOI:** 10.1007/s00018-022-04326-x

**Published:** 2022-06-04

**Authors:** J. S. Witteveen, S. R. Loopstok, L. Luque Ballesteros, A. Boonstra, N. H. M. van Bakel, W. H. P. van Boekel, G. J. M. Martens, J. E. Visser, S. M. Kolk

**Affiliations:** 1 Department of Molecular Animal Physiology, Donders Center for Neuroscience, Radboud University Nijmegen, Nijmegen, The Netherlands; 2 Department of Molecular Neurobiology, Donders Center for Neuroscience, Radboud University Nijmegen, Nijmegen, The Netherlands; 3 Department of Neurology, Donders Institute for Brain, Cognition and Behaviour, Radboud University Medical Center, Nijmegen, The Netherlands; 4 Department of Neurology, Amphia Hospital, Breda, The Netherlands

**Keywords:** HPRT1, Ventral tegmental area (VTA), Substantia nigra (SN), Radial glia, Otx2, Sox6

## Abstract

In Lesch–Nyhan disease (LND), deficiency of the purine salvage enzyme hypoxanthine guanine phosphoribosyl transferase (HGprt) leads to a characteristic neurobehavioral phenotype dominated by dystonia, cognitive deficits and incapacitating self-injurious behavior. It has been known for decades that LND is associated with dysfunction of midbrain dopamine neurons, without overt structural brain abnormalities. Emerging *post mortem* and in vitro evidence supports the hypothesis that the dopaminergic dysfunction in LND is of developmental origin, but specific pathogenic mechanisms have not been revealed. In the current study, HGprt deficiency causes specific neurodevelopmental abnormalities in mice during embryogenesis, particularly affecting proliferation and migration of developing midbrain dopamine (mDA) neurons. In mutant embryos at E14.5, proliferation was increased, accompanied by a decrease in cell cycle exit and the distribution and orientation of dividing cells suggested a premature deviation from their migratory route. An abnormally structured radial glia-like scaffold supporting this mDA neuronal migration might lie at the basis of these abnormalities. Consequently, these abnormalities were associated with an increase in area occupied by TH^+^ cells and an abnormal mDA subpopulation organization at E18.5. Finally, dopaminergic innervation was disorganized in prefrontal and decreased in HGprt deficient primary motor and somatosensory cortices. These data provide direct in vivo evidence for a neurodevelopmental nature of the brain disorder in LND. Future studies should not only focus the specific molecular mechanisms underlying the reported neurodevelopmental abnormalities, but also on optimal timing of therapeutic interventions to rescue the DA neuron defects, which may also be relevant for other neurodevelopmental disorders.

**Supplementary Information:**

The online version contains supplementary material available at 10.1007/s00018-022-04326-x.

## Introduction

Lesch–Nyhan disease (LND) is a disabling metabolic disorder with a characteristic neurobehavioral phenotype, dominated by generalized dystonia, executive cognitive deficits and incapacitating self-injurious behavior [1–3]. LND is caused by a mutation in the *HPRT1* gene, encoding the purine salvage enzyme hypoxanthine–guanine phosphoribosyl transferase (HGprt) [1, 2]. Despite the causal role of HGprt in LND was identified over half a century ago, the pathogenic mechanisms by which deficiency of this universally expressed enzyme leads to such specific motor and behavioral abnormalities are still incompletely understood.

Previous studies, including *post mortem* analysis of human brain tissue [4, 5], biochemical analysis of cerebrospinal fluid of LND patients [6] and positron emission tomography (PET) imaging in patients [7, 8] suggest that HGprt deficiency is associated with a selective reduction in dopamine (DA) levels in the brain’s basal ganglia. These findings are supported by results from in vitro HGprt deficient cell [9] and rodent models [10–12]. DA is essential in the development and functioning of multiple neural brain circuits involving midbrain, basal ganglia and cortical areas contributing to motor, cognitive and motivational aspects of behavior. It is thought that dysfunction of these circuits underlies the complex LND neurobehavioral phenotype [13]. Because the reduced DA levels in HGprt-deficient human or adult mouse midbrain regions cannot be explained by a loss of DA neurons [3, 5, 11, 14, 15], it has been suggested that HGprt deficiency causes a change in integrity of the dopaminergic neurochemical phenotype [14]. In fact, emerging evidence supports the hypothesis that the DA deficiency in LND is of developmental origin. First, the expression of a variety of developmental transcription factors (TFs) involved in DA system development is affected in multiple experimental HGprt-deficient cell models [9, 16–19]. Second, the disruption of these developmental molecular pathways is accompanied in vitro by aberrant expression of DA biosynthetic genes including tyrosine hydroxylase (TH) and aromatic l-amino acid decarboxylase (AADC), as well as vesicular monoamine transporter 2 (VMAT2) [20]. Third, differentiating HGprt-deficient neuroblastoma cells have shown an abnormal neurite outgrowth [17]. Fourth, both white and gray matter volumes are decreased in LND patients but are not accompanied by any other consistent structural pathology, suggesting maldevelopment rather than a degenerative process [21].

Development of midbrain DA (mDA) neurons is an intricate process, as mDA neurons constitute a highly heterogeneous cell population, organized in anatomically and functionally distinct nuclei [22]. Most of our knowledge about mDA system development has been obtained from studies in mice, where most of mDA neurogenesis occurs between embryonic days (E) 10.5 and E14.5 within the ventricular zone (VZ) of the floorplate of the midbrain [23–25]. A specific spatiotemporal expression pattern of signalling molecules and TFs establishes a mDA progenitor domain and regulates the generation of the presumptive substantia nigra (SN) mDA neurons followed by those of the presumptive ventral tegmental area (VTA) [26]. The early mDA progenitors show a close resemblance to the radial glia (RG) cells well described for the cerebral cortex and are referred to as radial glia-like (RG-L) cells [27, 28]. Following neurogenesis, developing mDA neurons migrate radially along processes of RG-L cells, from VZ towards the marginal zone (MZ) [25, 28–30]. Then, neurons destined for the SN migrate tangentially to a lateral position, while the presumptive VTA neurons remain medially [30–32]. In their final location, both SN and VTA mDA neurons will extend their axons towards their distant targets, receive efferent connections and further develop their dopaminergic neurochemical phenotype [25].

In the present study, we examined the effect of HGprt deficiency on early development of the dopaminergic midbrain in a genetic mouse model for LND—for the first time in vivo. We focused on proliferation and migration patterns of developing mDA neurons at two embryonic time points. In addition to abnormal numbers of cell division and altered spatial distribution of developing mDA neurons, we demonstrate that HGprt-deficiency is associated with an abnormal organization of the RG-L fiber scaffold to support mDA migration, with differences along the rostro-caudal axis. Consequently, these neurodevelopmental changes affected final SN and VTA subpopulation development and organization, as well as dopaminergic innervation of the cerebral cortex. This neurodevelopmental basis of disorganization of dopaminergic circuitry may contribute to the cognitive and behavioral phenotype of LND patients.

## Materials and methods

### Animals and tissue preparation

Wild-type *Hprt1*^*0/*+^ and congenic *HGprt*^*0/−*^ mutant mice were used, bred on a C57BL/6J background (*C57BL/6JHPRT*^*BM3*^*,* IMSR Cat# ORNL:B6.129P2-Hprt, RRID:IMSR_ORNL:B6.129P2-Hprt). As the *Hprt1* gene is X-linked, *Hprt1*^*0/−*^ male animals and *Hprt1*^*0/*+^ littermate controls were generated by mating wild-type males with heterozygous females. *Hprt1*^*0/−*^ mutant mice do not exhibit self-injurious behavior or overt dystonia like humans with LND, but they show abnormal behavioral sensitivity to amphetamine and abnormal open-field motor activity [33, 34].

The mice were housed in filter top Makrolon cages in a temperature- and humidity-controlled room (21 ± 1 °C and 60% relative humidity). Animals had ad libitum access to food and water and were kept at a 12:12 h light:dark cycle. All experiments were performed in accordance with the institutional, national and European ethical guidelines and regulations and approved by the Committee for Animal Experiments of the Radboud University Nijmegen, The Netherlands (RU-DEC 2011-305).

Mice were allowed to mate between 4 pm and 10 am, to prevent any ambiguity regarding timing of pregnancy. The day after mating was considered embryonic day (E)0.5 and the day of birth as postnatal day (P)0. Timed-pregnant mice were sacrificed by means of cervical dislocation. Embryos were dissected in ice-cold Leibovitz medium (L-15, PAA). Embryonic heads (E14.5) and embryonic brains (E18.5) were fixed by immersion in 4% paraformaldehyde (PFA) in phosphate buffered saline (PBS; 0.08 M Na_2_HPO_4_, 1.36 M NaCl, 0.017 M KH_2_PO_4_, 0.026 M KCl), pH 7.4, at 4 °C for 1–1.5 h. After a quick wash in PBS, brains were cryoprotected overnight in 30% sucrose (wt/vol) in PBS. Brains were frozen in M-1 embedding matrix (Thermo Fisher Scientific) in a plastic cup on dry ice and stored at −80 °C. Cryostat sections were cut at 16 μm, mounted on SuperFrost Plus slides (Thermo Fisher Scientific), air-dried and stored desiccated at −20 °C. Sex and *Hprt1* genotype of the animals were identified by polymerase chain reaction (PCR) applied to a DNA sample isolated from a tail clip (embryos) or ear punch (postnatal animals). Primer sequences are listed in Supplementary Table 1.

### Proliferation and migration assays

To assess proliferation and migration parameters at multiple time points, timed-pregnant mothers received one of the following two treatments. One group of pregnant mothers received an intraperitoneal (i.p.) injection of 5-bromo-2′-deoxyuridine (BrdU; BD biosciences, 50 µg/g) at E12.5, and was sacrificed at E14.5. Another group of pregnant mothers received an i.p. injection of 5-ethynyl-2´-deoxyuridine (EdU; Click-iT-Alexa Fluor555 Molecular Probes, 30 µg/g) at E16.5 and was sacrificed at E18.5. Embryonic heads or brains were dissected, fixed, cryoprotected and sectioned as explained above*.*

### Immunohistochemistry

Sections were rehydrated in PBS. Sections were then incubated in a normal blocking solution (NBS; 1.6% normal goat serum, 1.6% normal donkey serum, 1.6% normal horse serum, 1% BSA, 0.1% glycine, 0.1% lysine, 0.4% Triton X-100) for 1 h at room temperature (RT). Primary antibodies, listed in Supplementary Table 2, were diluted in NBS and incubated overnight at 4 °C. Slides were washed three times in PBS for a total of 30 min at RT. Sections were incubated with species-specific Alexa-conjugated secondary antibodies (Molecular Probes, Thermofisher) diluted in NBS for 1 h at RT. After three washes in PBS, 10 min each at RT, sections were incubated with blue fluorescent Nissl stain (NeuroTrace, Invitrogen; 1:500) or 4,6-diamidino-2-phenylindole (DAPI; Molecular Probes; 1:1000) for 15 min and washed three times in PBS; 10 min each at RT. Slides were mounted in 90% glycerol in PBS and stored at 4 °C.

For BrdU staining, cryosections were incubated with NBS for 1 h at RT and incubated with chicken anti-TH and rabbit anti-Ki67 diluted in NBS overnight at 4 °C. Cryosections were postfixed for 10 min in 4% PFA, followed by a quick wash step in PBS. Subsequently, sections were treated with 0.1% trypsin in 0.1% CaCl_2_ in 0.1 M Tris for 12 s, incubated in 100% fetal bovine/calf serum for 10 min and rinsed in PBS. Sections were incubated in 2 N HCl for 30 min at 37 °C with agitation, the acid was neutralized in 0.1 M sodium borate pH 8.5 and quickly washed in PBS. Standard immunohistochemistry was then performed as described above.

EdU staining was performed according to the manufacturer’s protocol. In short, cryosections were incubated with NBS for 1 h at RT, followed by incubation with the EdU reaction buffer (4% CuSO_4_, 0.25% Alexa Fluor Azide, 10% reaction buffer additive in Click-iT reaction buffer) for 30 min at RT. After a quick rinse with PBS, standard immunohistochemistry was then performed as described above.

### Imaging

All stained sections were visualized and images were captured using a Leica DMRA fluorescence microscope coupled with a DFC340FX digital camera or a Leica DMI6000B inverted microscope with DFC360FX camera, both using accompanying LASAF software, or with an Invitrogen/Thermo Fisher Scientific EVOSTM FL Auto Imaging System with high-sensitivity CMOS camera and EVOS FL Auto Software.

### Data analysis and statistics

Data quantification was performed by counters blinded to the genotype and expressed as an average ± SEM. For quantifications regarding the ventral midbrain, coronal sections were analyzed separately for rostral and caudal areas, based on the anatomical landmarks in the DA system as described previously [30, 32, 35, 36] and further outlined here (E14.5; Fig. 2A, E18.5; Fig. 3C). If quantification of rostral vs caudal groups showed no significant difference, the quantifications were combined and expressed in one graph under the denomination ‘total’.

**Fig. 1:**
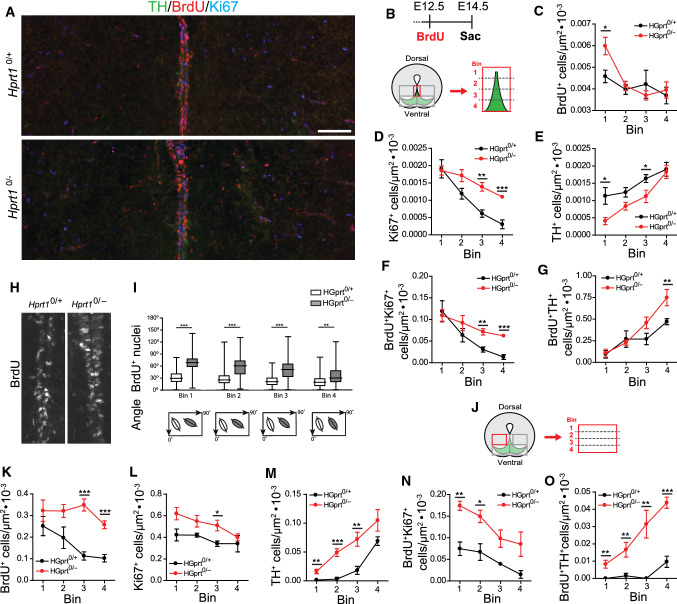
HGprt deficiency is associated with an increase in cell division and an abnormal cell alignment in the DA progenitor migration path. **A** E14.5 *Hprt1*^*0/*+^ and *Hprt1*^*0/−*^ embryo coronal brain sections containing the radial migration path and flanking areas in ventral midbrain, immunostained for TH (green), BrdU (red) and Ki67 (cyan). Scale bar: 100 µm. **B** Top: Pregnant dams were injected i.p. at E12.5 with BrdU and embryos were sacrificed at E14.5. Bottom: Schematic of the area investigated and enlargement of the binned area quantified as depicted in (**A**). **C–G** Quantification of BrdU^+^ cells (*Hprt1*^*0/*+^ /*Hprt1*^*0/−*^ *n* = 5/5; **C**), Ki67^+^ cells (*Hprt1*^*0/*+^ /*Hprt1*^*0/−*^ *n* = 3/4; **D**), TH^+^ cells (*Hprt1*^*0/*+^ /*Hprt1*^*0/−*^ *n* = 5/5; **E**), BrdU^+^Ki67^+^ cells (*Hprt1*^*0/*+^ /*Hprt1*^*0/−*^ *n* = 3/4; **F**) and BrdU^+^TH^+^ cells (*Hprt1*^*0/*+^ /*Hprt1*^*0/−*^ *n* = 5/5; **G**) in bins depicted in (**B**). Graphs represent average normalized number of positive cells (per µm^2^) for each of the 4 bins ± S.E.M. **H** E14.5 *Hprt1*^*0/*+^ and *Hprt1*^*0/−*^ embryo coronal brain sections containing the radial migration stream in ventral midbrain, immunostained for BrdU. **I** Quantification of the angle of BrdU^+^ nuclei with the midline within the radial migration path (*Hprt1*^*0/*+^ /*Hprt1*^*0/−*^ n = 5/5). Plots represent the average angle of nuclei for each of the 4 bins ± S.E.M. Schematic plots underneath illustrate the average angle of nuclei per bin. **J** Schematic of the area investigated and enlargement of the binned area quantified (bilateral) depicted in (**A**). **K–O** Quantification of BrdU^+^ cells (*Hprt1*^*0/*+^ /*Hprt1*^*0/−*^ *n* = 5/5; **K**), Ki67^+^ cells (*Hprt1*^*0/*+^ /*Hprt1*^*0/−*^ *n* = 3/4; **L**), TH^+^ cells (*Hprt1*^*0/*+^ /*Hprt1*^*0/−*^ *n* = 3/4; **M**), BrdU^+^Ki67^+^ cells (*Hprt1*^*0/*+^ /*Hprt1*^*0/−*^ *n* = 5/5; **N**) and BrdU^+^TH^+^ cells (*Hprt1*^*0/*+^ /*Hprt1*^*0/−*^ *n* = 5/5; **O**) in bins depicted in (**J**). Graphs represent average normalized number of positive cells (per µm^2^) for each of the 4 bins ± S.E.M. All statistical tests were performed using one-way ANOVA (*α* = 5%), *P < 0.05, **P < 0.01, ***P < 0.001

**Fig. 2:**
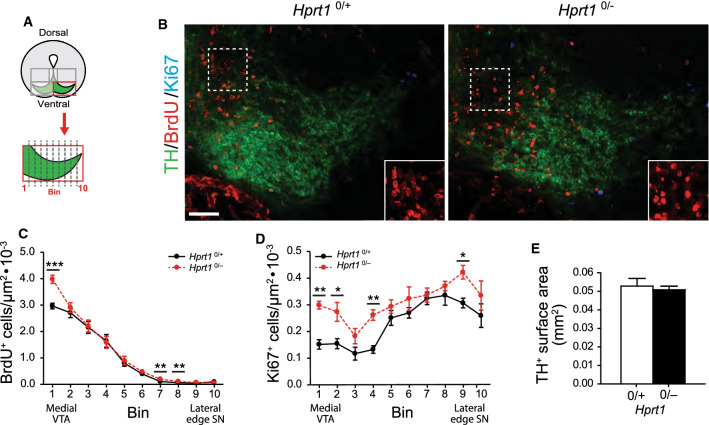
HGprt deficiency is associated with increased proliferation and an apparent disorganization. in the presumptive VTA and SN area. **A** Schematic of area investigated and enlargement of the binned area quantified depicted in (**B**). **B** E14.5 *Hprt1*^*0/*+^ and *Hprt1*^*0/−*^ coronal embryo brain sections containing the ventral midbrain, immunostained for TH (green), BrdU (red) and Ki67 (cyan). Scale bar: 50 µm. Boxed area is enlarged in the bottom right corner. **C**, **D** Quantification of BrdU^+^ cells (*Hprt1*^*0/*+^ /*Hprt1*^*0/−*^ *n* = 5/5; **C**) and Ki67^+^ cells (*Hprt1*^*0/*+^ /*Hprt1*^*0/−*^ *n* = 3/4; **D**) in bins depicted in (**A**). Graphs represent average normalized number of positive cells (per µm^2^) for each of the 10 bins ± S.E.M. **E** Quantification of the surface area containing TH^+^ cells (*Hprt1*^*0/*+^ /*Hprt1*^*0/−*^ n = 5/5). Graphs represent average surface area (mm^2^) ± S.E.M. All statistical tests were performed using one-way ANOVA (*α* = 5% ), *P < 0.05, **P < 0.01, ***P < 0.001

For quantification of labelled cell numbers in the ventral midbrain (E14.5 *Hprt1*^*0/*+^ *n* = 3–5, *Hprt1*^*0/−*^ *n* = 3–5; E18.5 *Hprt1*^*0/*+^ *n* = 3, *Hprt1*^*0/−*^ *n* = 3), the area was subdivided into three regions: the radial migration path (Fig. 1B), the lateral flanking areas of the migration path (Fig. 1J) and the early presumptive DA ventral midbrain (Fig. 2A). The radial migration path was divided into four equal bins of 0.1 mm width along the dorsoventral axis, with bin 1 located at the VZ. The flanking area on each side was similarly divided. The ventral midbrain was divided into 10 equal bins along the medio-lateral axis; with bin 1 located at the midline of the VTA and bin 10 at the most lateral edge of the presumptive SN. All cells were counted per bin and normalized to bin size, using ImageJ software (NIH, Bethesda, USA) and Adobe Photoshop software. Normalized cell numbers were subsequently averaged for the right and left side per section and finally averaged over sections per embryo and then per genotype. The TH^+^ surface area in the early ventral DA midbrain (E14.5 *Hprt1*^*0/*+^ n = 3, *Hprt1*^*0/−*^ *n* = 3; E18.5 *Hprt1*^*0/*+^ *n* = 3, *Hprt1*^*0/−*^ *n* = 3) was quantified by outlining the area containing TH^+^ cells using ImageJ software (NIH, Bethesda, USA). The data were tested for significance by one-way ANOVA (*α* = 5%) and expressed as means ± S.E.M.

For quantification of the angle of the nuclei of migrating neurons (E14.5 *Hprt1*^*0/*+^ *n* = 5, *Hprt1*^*0/−*^ *n* = 5), we determined the directional orientation of BrdU^+^ nuclei in the four bins of the radial migration path in 2–3 well-spaced coronal sections. Using ImageJ software, a line was drawn along the axis of the elongated nuclei and the angle of this line in relation to the midline of the radial migration path was measured. The angles were averaged per bin and were subsequently averaged for the right and left side per section and finally averaged over sections per embryo and then per genotype. The data were tested for significance by one-way ANOVA (*α* = 5%) and expressed as means ± S.E.M.

For analysis of the structural appearance of the RG-L process scaffold (E14.5 *Hprt1*^*0/*+^ n = 4, *Hprt1*^*0/−*^ *n* = 4), an overlay of 3 concentric circles, each subdivided in 4 zones (A–D), was placed over 2–3 coronal sections per region of the ventral midbrain (Fig. 2A, B). The number of intersections of RG cell marker-2-positive (RC2^+^) processes with these overlaid lines was counted per zone and normalized to number of intersections per µm. Normalized number of intersections were averaged across right and left side per section and subsequently averaged over sections per embryo and then per genotype. The integrity of the RG process scaffold within the cortical wall (E14.5 *Hprt1*^*0/*+^ *n* = 4, *Hprt1*^*0/−*^ *n* = 4) was measured in a swatch spanning the cortex by placing a rectangle of 100 µm width in the center of the cortical region of interest, in both hemispheres, in two well-spaced coronal sections. The number of RC2^+^ process intersections with a line drawn at the proliferative zone (PZ)/intermediate zone (IZ) border was quantified and normalized to the number of intersections per µm. Normalized number of intersections were averaged across right and left hemisphere per section and subsequently averaged over sections per embryo and then per genotype. The data were tested for significance by one-way ANOVA (*α* = 5%) and expressed as means ± S.E.M.

To examine the E18.5 cerebral cortex, a cortical swatch of 100 µm width in the center of the cortical region was analyzed. The cortical swatches were subsequently divided into 10 equal bins, with bin 1 located in the VZ and bin 10 in the MZ. To quantify the innervation of the cortical regions, the TH-positive (TH^+^) fibers were traced using ImageJ software plugin NeuronJ (NIH, Bethesda, USA). The total length per bin was normalized to bin size and averaged over sections per embryo and then per genotype. The data were tested for significance by one-way ANOVA (*α* = 5%) and expressed as means ± S.E.M.

## Results

### HGprt deficiency is accompanied by increased cell division and an abnormal cell alignment in the midline DA progenitor migration path during early development

Timed proliferation and migration events are fundamental for the proper development and organization of the mDA neuronal clusters. To investigate the effect of HGprt deficiency on proliferation and migration of TH^+^ mDA progenitors in the ventral midbrain between E12.5 and E14.5 we examined the brains of male HGprt-deficient (*Hprt1*^*0/−*^) embryos and their wild-type (*Hprt1*^*0/*+^) littermate controls. Proliferating cells were labelled by administration of BrdU, a thymidine analogue that is incorporated during the S-phase of the cell cycle [37] by i.p. injection of pregnant mothers two days before sacrifice (Fig. 1B). Visualization of the BrdU-positive (BrdU^+^) cells allowed us to distinguish between proliferating progenitors and post-mitotic populations, while their location provided information about their migratory efforts. A co-staining with the proliferation marker Ki67 was performed to examine which of the BrdU^+^ cells that were labelled after the injection at E12.5 were still in active cycle by E14.5 (Fig. 1A, B) [38].

Presumptive SN and VTA mDA neurons are initially generated in the VZ of the ventral midbrain from where they migrate towards the ventral MZ, forming a radial migration path [30, 31, 39]. Quantifying the number of the labelled cells in 4 equal bins along this dorsal–ventral axis (Fig. 1B) provided information about their distribution and revealed a significant increase in the number of BrdU^+^ cells in the bins near the VZ of *Hprt1*^*0/−*^ embryos as compared to their controls (Fig. 1C), as well as an increase in Ki67-positive (Ki67^+^) cells in the more ventral bins (Fig. 1D). The number of BrdU^+^/Ki67^+^ cells, i.e., cells that were in cell cycle at E12.5 and were still in cell cycle by E14.5, also showed a significant increase in these more ventral bins (Fig. 1F). Opposite to the increase in the number of proliferating BrdU^+^ cells, the number of TH^+^ cells was decreased particularly near the VZ in the midbrain of *Hprt1*^*0/−*^ embryos, but this effect diminished further along the radial migration path (Fig. 1E). Of note, the number of BrdU^+^/TH^+^ cells remained unaltered close to the VZ as well and increased more ventrally in the radial migration path (Fig. 1G). Rostral and caudal areas appeared to be affected equally. Taken together, these findings portray an increase in proliferation within the ventral midbrain in *Hprt1*^*0/−*^ embryos between E12.5 and E14.5, accompanied by an altered distribution of proliferating cells that remain in cell cycle, as well as a decrease in presumptive dopaminergic cells at this developmental stage.

In addition to these observed deviations in cell numbers, an abnormal alignment along the midline of the radial migration path was visible, evident from the orientation of the BrdU^+^ nuclei (Fig. 1H). Generally, migrating neurons are characterized by a polarized profile, with their nuclei elongated and oriented with the longer axis in the direction of migration [40, 41]. In controls, a large portion of the BrdU^+^ nuclei were visibly elongated and positioned in the direction of the radial migration path (Fig. 1A, H, I). In the *Hprt1*^*0/−*^ embryos however, elongated BrdU^+^ nuclei were positioned in a noticeably different angle, deviating from the direction of the migration path, with the most pronounced difference at the start of the migration path, close to the VZ (Fig. 1A, H, I). The majority of the elongated BrdU^+^ nuclei were Ki67^−^, indicating it probably concerns the differentiated cell population (data not shown).

To explore whether this altered progenitor alignment in *Hprt1*^*0/−*^ embryos could indicate an abnormal direction of mDA progenitor migration, we inspected the areas flanking the radial migration path (Fig. 1A, J). Here we observed an increase in the number of BrdU^+^, Ki67^+^ and BrdU^+^/Ki67^+^ cells in multiple bins quantified in *Hprt1*^*0/−*^ compared to control embryos (Fig. 1K, L, N). We additionally observed a notable increase in the number of TH^+^ cells (Fig. 1M), as well as BrdU^+^/TH^+^ cells in the areas lateral to the radial migration path of *Hprt1*^*0/−*^ embryos (Fig. 1O). These results suggest that mDA progenitors are indeed prematurely deviating from the radial migration path in the absence of HGprt.

Once the presumptive SN and VTA mDA neurons have reached the MZ of the ventral midbrain, the SN mDA neurons will change migratory bearing from radial into a tangential direction towards lateral regions, while the VTA mDA neurons remain in the medial area [30, 32]. When comparing this area of *Hprt1*^*0/−*^ embryos with controls at E14.5, an apparent disorganization was observed. First, the *Hprt1*^*0/−*^ BrdU^+^ nuclei appeared overall more rounded and not elongated in the direction of the migratory path as in control embryos (Fig. 2A, B inset). In addition, BrdU^+^ cell clusters could be seen in the *Hprt1*^*0/−*^ in this area. In order to quantify this, the area containing the presumptive SN and VTA was divided in 10 equal bins along the medio-lateral axis (Fig. 2A). Quantification revealed an increase in BrdU^+^ cells, particularly around the VTA midline, as well as an overall increase in Ki67^+^ cells (Fig. 2C, D). There were almost no BrdU^+^/Ki67^+^ cells found in this area for either genotype (data not shown). The close proximity of the TH^+^ cells at this developmental time point made it difficult to distinguish individual TH^+^ cells, hence a TH^+^ surface area was quantified instead. This, however, was not affected by HGprt deficiency (Fig. 2E). Taken together, these data show that HGprt deficiency is associated with increased proliferation and altered migration patterns in the DA ventral midbrain at E14.5.

### HGprt deficiency is associated with changes in RG-L process scaffolding in the ventral midbrain

**Fig. 3:**
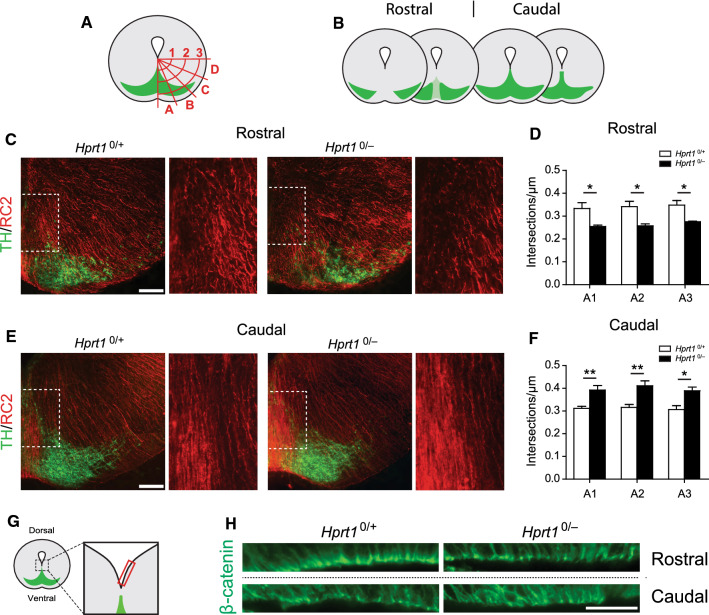
Changes in radial glia-like scaffolding in HGprt-deficient ventral midbrains. **A** Schematic of the area investigated and area quantified; overlay of three concentric circles (1–3) subdivided into four zones (**A**–**D**). Intersections of RC2^+^ processes with the overlay lines were counted. **B** Schematic of coronal brain sections containing the SN and VTA in the rostro-caudal axis in E14.5 mouse brain. Rostral and caudal sections were examined separately. **C**, **D** Representative images of coronal brain sections of the rostral DA area (**C**) and the caudal DA area (**D**) of the ventral midbrain of E14.5 *Hprt1*^*0/*+^ and *Hprt1*^*0/−*^ embryos, immunostained for RC2 (red) and TH (green). Scale bar: 100 µm. Boxed area is enlarged on the right. (**E, F**) Quantification of RC2^+^ process intersections with overlay in rostral (**E**) and caudal (**F**) sections (*Hprt1*^*0/*+^ /*Hprt1*^*0/−*^ *n* = 4/4). Graphs represent average normalized number of RC2^+^ process intersections (µm) per line in zone A ± S.E.M. **G** Schematic of area investigated (red rectangle) as depicted in (**H**) with the DA neurons in green. **H** Representative images of rostral and caudal coronal sections of the VZ close to the midline of the ventral midbrain of E14.5 *Hprt1*^*0/*+^ and *Hprt1*^*0/−*^ embryos, immunostained for β-catenin (green) Scale bar: 25 µm. All statistical tests were performed using one-way ANOVA (*α* = 5% ), *P < 0.05, **P < 0.01

During midbrain development, RG-L cells of which the cell bodies are located primarily in the VZ play a dual role. First, they undergo apical neurogenic divisions generating mDA precursor cells. Second, their glial-processes provide a scaffold running from the VZ towards the pial surface to support and guide cellular migration [25, 27–29, 42]. The observed changes in proliferation profile with a possible preliminary deviation from the radial migration path in the E14.5 *Hprt1*^*0/−*^ embryos led to the hypothesis that HGprt deficiency affects the RG-L cell population and subsequently their glial process scaffold. The number and organization of RG cell marker-2-positive (RC2^+^) processes throughout the ventral midbrain of E14.5 *Hprt1*^*0/−*^ embryos and their controls was assessed [43]. Using an overlay of concentric circles on the ventral midbrain, subdivided in multiple zones (Fig. 3A), the number of intersections of the RG-L processes with these circles was quantified, to assess the structural organization of this scaffold. As the rostral and caudal ventral midbrain sections appeared differentially affected by the HGprt deficiency, both regions were analyzed separately (Fig. 3B).

The number of RC2^+^ processes, in both rostral and caudal sections, appeared to be affected solely in the zone containing the midline of the ventral midbrain (Sect. A1-A3; Fig. 3A), while no differences were found in the more lateral zones (Supplemental Fig. 1A, B). Of note, the number of RC2^+^ process intersections in the rostral sections was significantly reduced (Fig. 3C, E) while they were inversely affected in the more caudal sections (Fig. 3D, F). In addition, in the control embryos the RC2^+^ processes appeared as taut processes, while in the *Hprt1*^*0/−*^ embryos the processes appeared more disorganized (Fig. 3C, D, enlargement of the boxed area).

The characteristic bipolar radial morphology of the RG-L cells allows two locations of adhesion: the end feet of the basal RG-L processes attached to the subpial extracellular matrix through integrin–laminin interactions [44, 45] and the apical RG-L end feet attached via adherence junctions within the VZ [46]. Adherence junctions are highly adhesive complexes consisting of multiple components including β-catenin, important for the integrity of the scaffold, including the polarity of the RG-L cells [47]. To further assess the integrity of this scaffold, we examined the β-catenin expression patterns in the VZ of the area close to the midline in E14.5 *Hprt1*^*0/−*^ embryos and their controls (Fig. 3G, H). In rostral sections, there appeared to be consistently fewer β-catenin-positive (β-catenin^+^) structures in the VZ close to the midline of the ventral midbrain in *Hprt1*^*0/−*^ embryos compared to their controls. Reversely, in caudal sections there appeared to be an increase in β-catenin^+^ structures in the *Hprt1*^*0/−*^ embryos (Fig. 3H). This is in agreement with the direction of change demonstrated in the number of the RG-L processes (Fig. 3D, F). Also, the organization of the β-catenin^+^ structures was markedly different between genotypes, with a more tousled arrangement observable in the *Hprt1*^*0/−*^ embryos (Fig. 3H). Taken together, these data suggest an altered organization and integrity of the apical end feet of the RG-L cells in absence of HGprt.

The principle of a migration-facilitating RG process scaffold has been extensively described in frontal cortical regions [48, 49]. To explore whether the HGprt deficiency associated effect on this scaffold was restricted to the ventral midbrain or could also be found in other brain regions, the experiment was repeated in several DA cortical target regions; the three sub-regions of the medial prefrontal cortex (mPFC) [i.e., the infralimbic cortex (IL), the prelimbic cortex (PL) and the cingulate cortex (CG)], the somatosensory cortex (S1) and the motor cortex (M1). To this extent the number of intersections of RG processes with a line positioned at the border of the proliferative zone (PZ) with the intermediate zone (IZ) was quantified in a 100 µm wide cortical swatch placed in the center of the cortical region of interest. At E14.5 there were no changes found in the RG processes in any of the examined cortical regions in *Hprt1*^*0/−*^ embryos compared to their controls (Supplemental Fig. 1C, D). This suggests that the abnormalities found in the RG process scaffold in the ventral midbrain in *Hprt1*^*0/−*^ embryos are either region-specific affecting only the midbrain, time-specific affecting the midbrain at embryonic stage E14.5 but other brain regions possibly at other developmental time points, or both.

### Increased cell divisions do not affect the number of TH^+^ cells at a later developmental stage

**Fig. 4:**
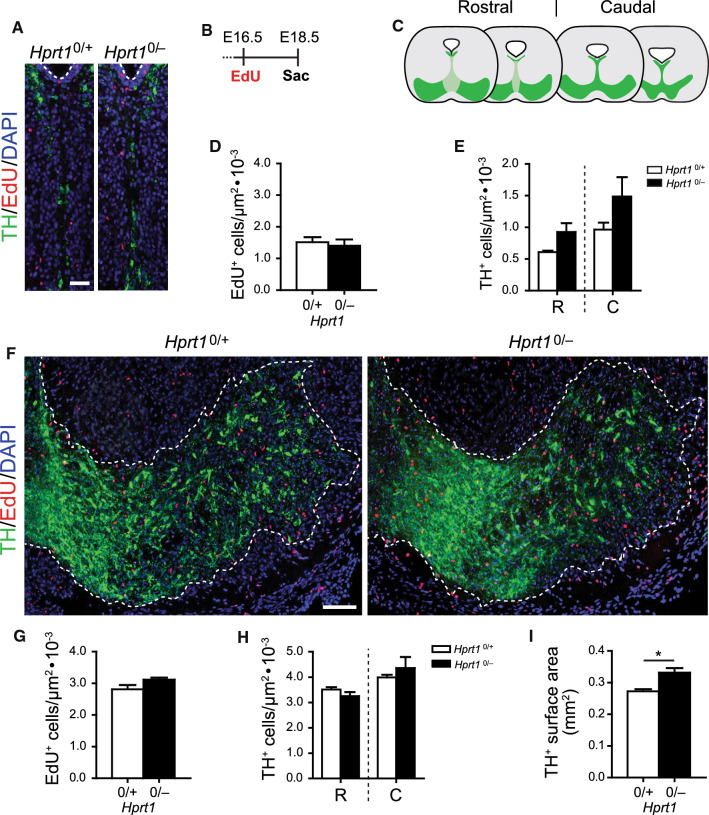
Increased cell divisions in HGprt-deficient ventral midbrains do not affect number of TH^+^ cells at later developmental stage. **A** Representative images of coronal brain sections containing the radial migration path in the ventral midbrain of E18.5 *Hprt1*^*0/*+^ and *Hprt1*^*0/−*^ embryos, immunostained for TH (green), EdU (red) and Ki67 (cyan), counterstained with DAPI (blue). Scale bar: 25 µm. **B** Pregnant mice were injected i.p. at E16.5 with EdU and embryos were sacrificed at E18.5. **C** Schematic of coronal brain sections containing the SN and VTA (green) in the rostro-caudal axis in E18.5 mouse brain. Rostral and caudal sections were examined separately. **D** Quantification of total number of EdU^+^ cells (*Hprt1*^*0/*+^ /*Hprt1*^*0/−*^ *n* = 3/3) in area depicted in (**A**). Graphs represent average normalized number of EdU^+^ cells (per µm^2^) ± S.E.M. **E** Quantification of total number of TH^+^ cells (*Hprt1*^*0/*+^ /*Hprt1*^*0/−*^ *n* = 3/3) in area depicted in (**A**) for rostral and caudal sections separately. Graphs represent average normalized number of TH^+^ cells (per µm^2^) ± S.E.M. **F** Representative images of coronal brain sections containing the ventral midbrain of E18.5 *Hprt1*^*0/*+^ and *Hprt1*^*0/−*^ embryos, immunostained for TH (green), EdU (red) and counterstained with DAPI (blue). Scale bar: 100 µm. **G** Quantification of total number of EdU^+^ cells (*Hprt1*^*0/*+^ /*Hprt1*^*0/−*^ *n* = 3/3) in area depicted in (**F**). Graphs represent average normalized number of EdU^+^ cells (per µm^2^) ± S.E.M. **H** Quantification of total number of TH^+^ cells (*Hprt1*^*0/*+^ /*Hprt1*^*0/−*^ *n* = 3/3) in area depicted in (**F**) for rostral and caudal sections separately. Graphs represent average normalized number of TH^+^ cells (per µm^2^) ± S.E.M. **I** Quantification of surface area containing TH^+^ cells as depicted in (**F**, dotted line) (*Hprt1*^*0/*+^ /*Hprt1*^*0/−*^ *n* = 3/3). Graphs represent average surface area (mm^2^) ± S.E.M. All statistical tests were performed using one-way ANOVA (*α* = 5% ), *P < 0.05. *C* caudal, *R* rostral

The increased number of proliferating cells in the *Hprt1*^*0/−*^ DA midbrain between E12.5-E14.5, could be due to an extension of the cell cycle period causing a developmental delay. For that reason, we assessed the proliferation profile at a later developmental time point, by labelling proliferating cells with EdU, another analogue of thymidine [50], administered two days prior to sacrifice at E18.5 (Fig. 4B). Coronal sections were co-stained with TH, counter-stained with DAPI and cell numbers and distribution were quantified for rostral and caudal sections separately (Fig. 4C).

In the radial migration path, there were no differences observed in the total EdU-positive (EdU^+^) cell number (Fig. 4A, D) or distribution (Supplemental Fig. 2A), nor were there any differences between rostral and caudal sections (data not shown). In the area containing the presumptive SN and VTA, the total EdU^+^ cell number was also not affected (Fig. 4F, G), although there was an isolated significant increase in EdU^+^ cells close to the midline of the presumptive VTA in *Hprt1*^*0/−*^ embryos compared to controls (Supplemental Fig. 2C).

It could be speculated that the number or distribution of TH^+^ mDA neurons at E18.5 is affected by the proliferative and migratory abnormalities observed during earlier development in *Hprt1*^*0/−*^ embryos. However, only a modest nonsignificant increase in total TH^+^ cell number was found in the area containing the former radial migration path (Fig. 4E). Dividing this area in 4 equal bins revealed a pattern of TH^+^ cell distribution in *Hprt1*^*0/−*^ embryos comparable to controls, just with a slight increase in the more ventral bins (Supplemental Fig. 2B). A similar lack of major differences was seen in the ventral DA midbrain, where overall the TH^+^ cell numbers were similar in *Hprt1*^*0/−*^ embryos compared to controls, albeit a minimal effect of HGprt deficiency was observed only close to the midline of the rostral presumptive VTA (Fig. 4H, Supplemental Fig. 2D). Of note, this is the same area where the isolated increase in EdU^+^ cells was found. Virtually no EdU^+^ and TH^+^ double positive (EdU^+^/ TH^+^) cells were observed at this developmental time point (data not shown). Taken together, these findings in E18.5 HGprt*-*deficient embryos indicate that the proliferative profile as well as the increase in TH^+^ cells has returned to a condition virtually comparable to the controls. Only near the midline of the DA midbrain there is still an increase in proliferation and number of TH^+^ cells. Despite these minimal differences at E18.5 in cell numbers, quantification of the TH^+^ surface area revealed that the TH^+^ cells were occupying an enlarged area in the *Hprt1*^*0/−*^ embryos compared to their controls (Fig. 4I).

### HGprt deficiency affects dopaminergic subtype marker expression in SN as well as VTA

Each of the stages in mDA neurodevelopment is defined by a spatiotemporal controlled expression of TFs and other molecular markers. Previous research has identified multiple signalling and transcriptional networks that are important for mDA neuronal subtype diversification, including a selected number that have a restricted expression in either the SN or the VTA [23, 25]. Among those, SRY-box6 (Sox6), was identified as a key TF for the specification and development of SN mDA neurons, while the TF orthodenticle-homeobox 2 (Otx2) is important for controlling the subtype identity of VTA neurons [31]. To analyze whether the observed altered proliferation and migration patterns affected mDA neuronal subtype specification, we analyzed the number and distribution of Sox6-positive (Sox6^+^) and Otx2-positive (Otx2^+^) cells in coronal sections of the ventral midbrain of E14.5 and E18.5 *Hprt1*^*0/−*^ and control animals.

**Fig. 5:**
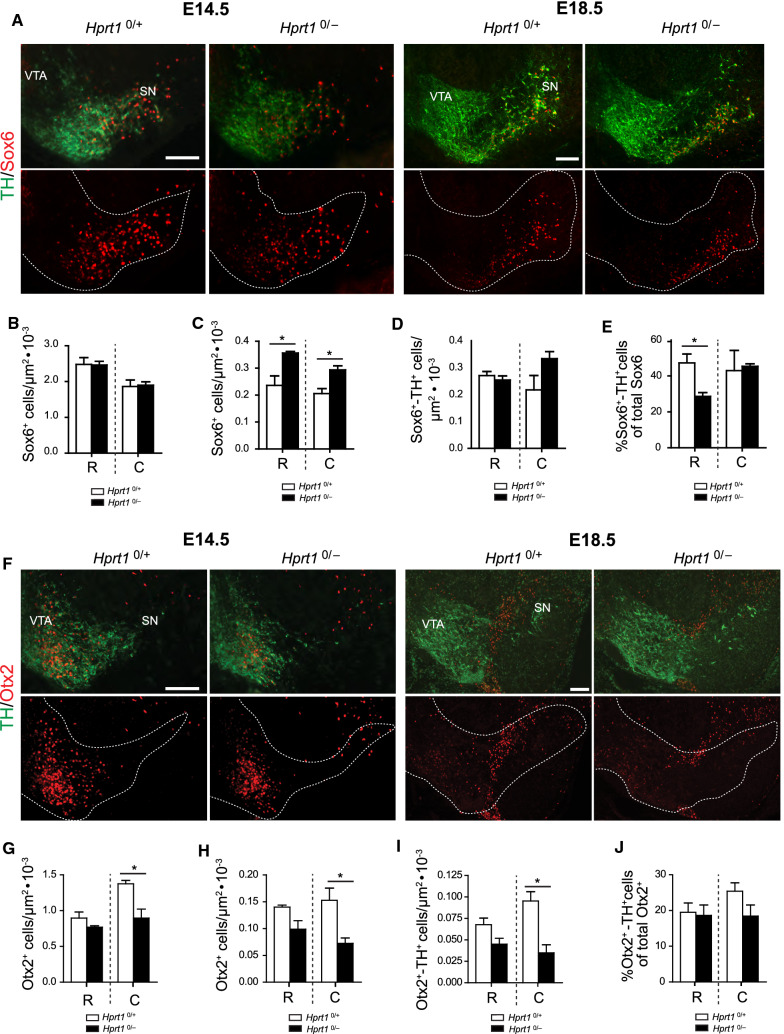
Altered distribution of Sox6^+^ and Otx2^+^ cells in developing HGprt-deficient ventral midbrain. **A** Examples of E14.5 and E18.5 *Hprt1*^*0/*+^ and *Hprt1*^*0/−*^ coronal brain sections containing the rostral ventral midbrain, immunostained for TH (green) and Sox6 (red). Panels underneath indicate Sox6^+^ cell distribution in ventral midbrain, dotted line indicates TH^+^ area. Scale bar: 100 µm. **B** Quantification of the total number of Sox6^+^ cells in E14.5 rostral and caudal sections (*Hprt1*^*0/*+^ /*Hprt1*^*0/−*^ *n* = 3/3). Graphs represent total normalized number of Sox6^+^ cells (per µm^2^) ± S.E.M. **C–E** Quantification of the total number of Sox6^+^ cells in rostral and caudal sections (*Hprt1*^*0/*+^ /*Hprt1*^*0/−*^ *n* = 3/3; **C**), the total number of Sox6^+^TH^+^ cells in rostral and caudal sections (*Hprt1*^*0/*+^ /*Hprt1*^*0/−*^ *n* = 3/3; **D**) and percentage of Sox6^+^TH^+^ cells of the total Sox6^+^ cell population in rostral and caudal sections (*Hprt1*^*0/*+^ /*Hprt1*^*0/−*^ n = 3/3; **E**). Graphs represent total normalized number of cells (per µm^2^) ± S.E.M. **F** Examples of E14.5 and E18.5 *Hprt1*^*0/*+^ and *Hprt1*^*0/−*^ coronal brain sections containing the caudal ventral midbrain, immunostained for TH (green) and Otx2 (red). Panels underneath indicate Otx2^+^ cell distribution in ventral midbrain, dotted line indicates TH^+^ area. Scale bar: 100 µm. **G** Quantification of the total number of Otx2^+^ cells in E14.5 rostral and caudal sections (*Hprt1*^*0/*+^ /*Hprt1*^*0/−*^ n = 3/3). Graphs represent total normalized number of Otx2^+^ cells (per µm^2^) ± S.E.M. **H–J** Quantification of the total number of Otx2^+^ cells in rostral and caudal sections (*Hprt1*^*0/*+^ /*Hprt1*^*0/−*^ *n* = 3/3; **H**), the total number of Otx2^+^TH^+^ cells in rostral and caudal sections (*Hprt1*^*0/*+^ /*Hprt1*^*0/−*^ *n* = 3/3; **I**) and percentage of Otx2^+^TH^+^ cells of the total Otx2^+^ cell population in rostral and caudal sections (*Hprt1*^*0/*+^ /*Hprt1*^*0/−*^ *n* = 3/3; **J**). Graphs represent total normalized number of cells (per µm^2^) ± S.E.M. All statistical tests were performed using one-way ANOVA (*α* = 5% ), *P < 0.05. **P < 0.01. *C* caudal, *R* rostral, *SN* substantia nigra, *VTA* ventral tegmental area

At E14.5, the Sox6^+^ cells in rostral sections of *Hprt1*^*0/−*^ embryos, indicating the presumptive SN mDA neurons, were distributed in a similar pattern as in the control sections, albeit their distribution had slightly shifted towards the midline, associated with incidental significant differences in the number of Sox6^+^ cells per bin (Fig. 5A, Supplemental Fig. 3A). On the other hand, the E14.5 HGprt-deficient Sox6^+^ cells in the caudal sections appeared shifted in the opposite direction compared to the rostral sections, i.e. towards lateral areas, with a significant decrease in the number of Sox6^+^ cells close to the midline (Supplemental Fig. 3B). As the total density of Sox6^+^ cells remained the same (Fig. 5B), it appears that particularly the distribution of these cells is affected by HGprt deficiency, both in rostral and caudal sections at E14.5.

At E18.5, a much more profound effect of the lack of HGprt on the number and distribution of Sox6^+^ cells in the ventral midbrain was noted; the mediolateral shift without difference in absolute cell count, as seen at E14.5, was not apparent anymore. However, at E18.5, there was a significant increase in Sox6^+^ cell numbers in the *Hprt1*^*0/−*^ compared to the control ventral midbrains (Fig. 5A, C), in both rostral and caudal sections. In the rostral sections, this increase was predominantly found close to the lateral edge of the presumptive SN, while in the caudal sections the increase in Sox6^+^ cells was confined to more central areas (Supplemental Fig. 3E, F). Additionally, in both rostral and caudal sections, the Sox6^+^ cells were located much more ventral within the TH^+^ area in *Hprt1*^*0/−*^ compared to the control ventral midbrains (Fig. 5A). Despite the increase in number of Sox6^+^ cells, the total number of Sox6 and TH double-positive (Sox6^+^/TH^+^) cells did not seem to be significantly affected by the absence of HGprt in both rostral and caudal sections, although there appears to be a slight increase double-positive cells in caudal areas when HGprt is absent (Fig. 5D, Supplemental Fig. 3G, H). Indeed, the significant reduction in the percentage of Sox6^+^/TH^+^ of the total Sox6^+^ pool found in the rostral sections in the absence of HGprt is not apparent in the caudal sections. These results suggest that, at this time point, particularly the Sox6^+^ cells did not fully acquire their dopaminergic phenotype yet, or that the Sox6^+^/TH^−^ pool is larger in mutants compared to controls (Fig. 5E, Supplemental Fig. 3I, J).

At E14.5, Otx2^+^ cells indicating the presumptive VTA mDA neurons, also showed abnormalities in number and distribution in *Hprt1*^*0/−*^ embryos that differed between rostral and caudal regions. Otx2^+^ cell numbers were significantly decreased only in the area close to the midline in *Hprt1*^*0/−*^ embryos in rostral sections compared to controls, but a more profound decrease was noticed in almost all bins containing the presumptive VTA in the caudal sections (Fig. 5F, Supplemental Fig. 3C, D). Otx2^+^ cell density confirmed this and showed a significant decrease in Otx2^+^ cells in the caudal sections of *Hprt1*^*0/−*^ embryos (Fig. 5G).

At E18.5, HGprt-deficiency had a similar effect on the number and distribution of Otx2^+^ cells in the ventral midbrain, albeit more pronounced. A decrease in Otx2^+^ cells was noticed, now more extending to the center of the VTA in the rostral sections (Fig. 5F, Supplemental Fig. 3K), while in caudal sections this decrease was present in virtually all bins containing the VTA (Fig. 5F, Supplemental Fig. 3L). Again, total Otx2^+^ cell counts confirmed this significant decrease in Otx2^+^ cell density (Fig. 5H). Moreover, the number of Otx2 and TH double-positive (Otx2^+^/TH^+^) cells, as a marker for cells with an established dopaminergic fate, was decreased in line with the total number of Otx2^+^ cells, that reached significance for the caudal areas (Fig. 5I, Supplemental Fig. 3M, N). As there was no difference to be observed in the fraction Otx2^+^/TH^+^ cells of total Otx2^+^ cells, it appeared that dopaminergic and nondopaminergic Otx2^+^ cells were equally affected (Fig. 5J and Supplemental Fig. 3O, P).

**Fig. 6:**
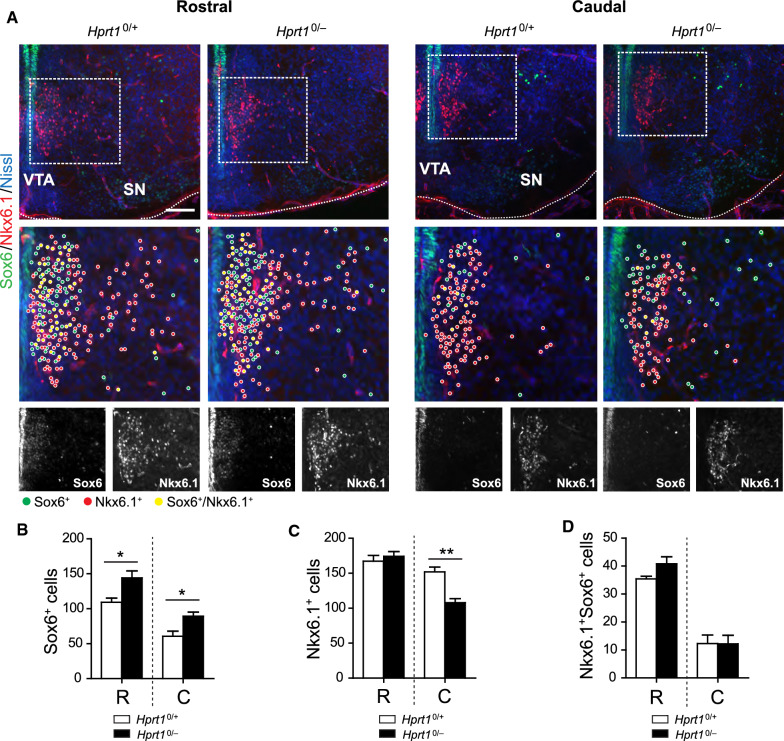
Sox6^+^ and Nkx6.1^+^ expression varies in the early ventral midbrain deficient of HGprt. **A** E14.5 *Hprt1*^*0/*+^ and *Hprt1*^*0/−*^ rostral and caudal coronal brain sections containing the ventral midbrain, immunostained for Sox6 (green), Nkx6.1 (red) and counterstained with Nissl (blue). Boxes show enlargement of Nkx6.1^+^ Sox6^+^ cell clusters flanking the radial migration path, followed by 8-bit images of the separate channels. Scale bar: 100 µm. **B**–**D** Quantification of Sox6^+^ cells (*Hprt1*^*0/*+^ /*Hprt1*^*0/−*^ *n* = 3/3; **B**), Nkx6.1^+^ cells (*Hprt1*^*0/*+^ /*Hprt1*^*0/−*^ *n* = 3/3; **C**) and Nkx6.1^+^ Sox6^+^ cells (*Hprt1*^*0/*+^ /*Hprt1*^*0/−*^ *n* = 3/3; **D**) in rostral and caudal sections. Graphs represent average number of positive cells ± S.E.M. All statistical tests were performed using one-way ANOVA (*α* = 5% ), *P < 0.05, **P < 0.01. *C* caudal, *R* rostral, *SN* substantia nigra, *VTA* ventral tegmental area

To investigate further into what extent these effects of HGprt deficiency affect also nondopaminergic (i.e. TH^−^) lineages in the developing ventral midbrain, we focused on a Sox6^+^/TH^−^ cell cluster flanking the radial migration path positive for homeobox protein Nkx6.1 (Nkx6.1^+^), a cell identity marker of the red nucleus and oculomotor neurons (Fig. 6A) [51]. We observed a significant increase in the number of Sox6^+^ cells in both rostral and caudal sections of *Hprt1*^*0/−*^ ventral midbrains, again with a distribution that appeared less confined than in controls (Fig. 6A, B). The number of Nkx6.1^+^ cells, however, was unaffected in the rostral sections and significantly reduced in the caudal sections of HGprt*-*deficient ventral midbrains (Fig. 6A, C). The total number of double-positive (Sox6^+^/Nkx6.1^+^) cells remained unaltered (Fig. 6D). Jointly, these data confirm the notion that the effects of HGprt deficiency on midbrain development are not restricted to the TH^+^ domain in the ventral midbrain. Whether these TH^−^ abnormalities are due to direct effects of HGprt deficiency on these lineages, or indirect via the abnormally patterned expression of Otx2 that is known to control the expression of Nkx6.1 [52], remains to be established.

### HGprt deficiency is associated with abnormal TH^+^ innervation of cortical areas

Based on the effects of HGprt deficiency on the proliferation and migration patterns shown here, it is hypothesized that these derangements may also influence the further development of the developing mDA neurons, in particular the axonal outgrowth and innervation of target areas. In fact, it has been hypothesized that dysfunction of these target areas due to altered dopaminergic innervation are important in the establishment of the clinical Lesch–Nyhan phenotype [13]. Ascending TH^+^ axons originating from the mDA neurons make precise synaptic connections with multiple target areas, including striatal and cortical areas [53, 54]. The architecture of cortical DA projections, predominantly formed by VTA mDA neurons, innervating different subdomains of the mPFC, i.e. infralimbic (IL), prelimbic (PL) and cingulate cortices (CG), has been well characterized [35, 55, 56]. In addition, the motor cortex (M1) and somatosensory cortex (S1) appear recipients of direct TH^+^ projections originating from the VTA [57, 58]. In order to assess the cortical TH^+^ innervation in an HGprt-deficient environment, coronal sections of E18.5 control and *Hprt1*^*0/−*^ brains containing the subdomains of the mPFC, the M1, and S1 were stained for TH, and TH^+^ axon density was measured.

**Fig. 7:**
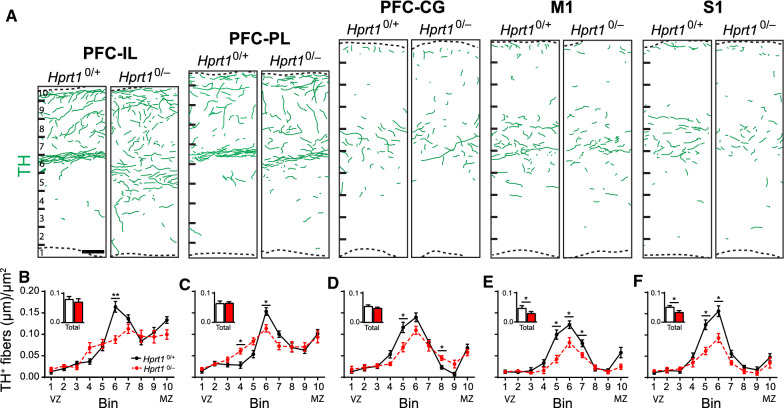
Effect of HGprt deficiency on innervation of frontal cortical regions. **A** Representative camera lucida drawings of swatches from coronal sections of the IL, PL, CG, M1 and S1 of E18.5 *Hprt1*^*0/*+^ and *Hprt1*^*0/−*^ embryos, immunostained for TH (green lines). Scale bar: 50 µm. **B–F** Quantification of TH^+^ fibers (*Hprt1*^*0/*+^ /*Hprt1*^*0/−*^ *n* = 3/3) in IL (**B**), PL (**C**), CG (**D**), M1 (**E**) and S1 (**F**). Graphs represent average normalized fiber length (µm/µm^2^) for each of the 10 bins ± S.E.M. Bar graph inserts represent the quantification of the total length of TH^+^ fibers (*Hprt1*^*0/*+^ /*Hprt1*^*0/−*^ n = 3/3) in the area depicted in (**A**). Graphs represent average normalized fiber length (µm/µm^2^) ± S.E.M. All statistical tests were performed using one-way ANOVA (*α* = 5% ), *P < 0.05, **P < 0.01. *CG* cingulate cortex, *IL* infralimbic cortex, *M1* motor cortex, *MZ* marginal zone, *PL* prelimbic cortex, *S1* somatosensory cortex, *VZ* ventricular zone

In the *Hprt1*^*0/−*^ E18.5 PFC subdomains, the total TH^+^ axon density was not altered (Fig. 7A, B–D inserts), but the distribution of the axons throughout the cortical wall was affected. In control mPFC subdomains (Fig. 7A), a noticeable organization of TH^+^ axons could be observed with the highest density in the marginal zone (MZ, corresponding to Bin 10 in Fig. 6) and the subplate (SP, Bin 6) and fewer TH^+^ axons invading the cortical plate (CP, Bin 6–9). In the *Hprt1*^*0/−*^ mPFC subdomains however, this organization was not as apparent and TH^+^ axon density appeared more evenly distributed throughout the cortical wall (Fig. 7A–D). In the HGprt*-*deficient M1 and S1 cortical regions, however, the total TH^+^ axon density did show a significant reduction, particularly in the SP region (Fig. 7A, E and F inserts), while the distribution of the TH^+^ axons remained comparable (Fig. 7A, E, F). In summary, these results demonstrate that HGprt deficiency not only affects the intrinsic development of the DA midbrain, but also involves the mDA innervation of multiple cortical target regions, by either affecting the spatial fiber organization at the cortical level (i.e., mPFC subdomains) or the fiber density (i.e., in M1 and S1).

## Discussion

**Fig. 8:**
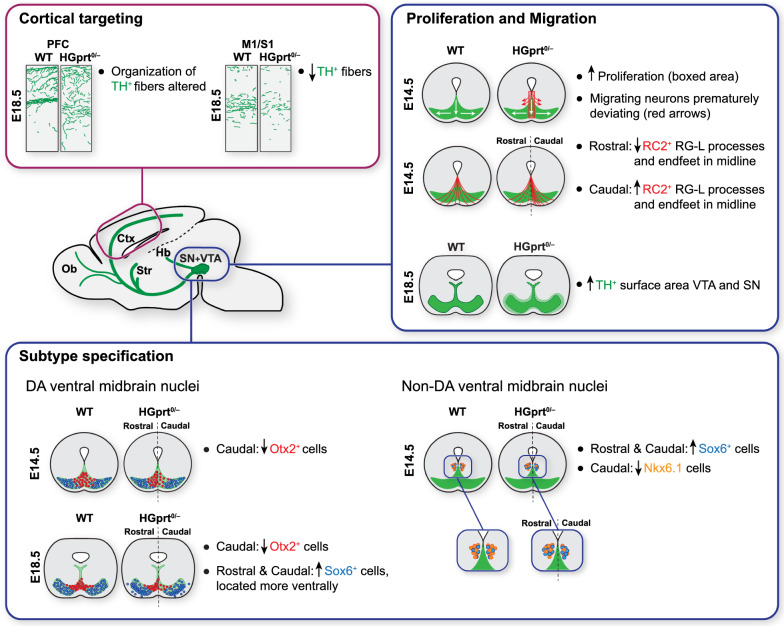
Schematic overview illustrating the main histological abnormalities found in *Hprt1*^*0/−*^ brains during development. *Ctx* cerebral cortex, *Hb* habenula, *M1* motor cortex, *Ob* olfactory bulb, *PFC* prefrontal cortex, *S1* somatosensory cortex, *SN* substantia nigra, *Str* striatum, *VTA* ventral tegmental area

A better insight in the pathogenic mechanisms of LND, in which a mutation in the *HPRT1* gene causes a complex neurobehavioral phenotype including dystonia, cognitive defects and self-injurious behavior, is crucial for developing new treatment opportunities for this incapacitating disease. Previous studies have not revealed specific neurodegenerative or other structural brain abnormalities, but indicate that otherwise healthy-appearing mDA neurons fail to express TH [59], resulting in reduced DA levels in dopaminergic brain areas [2, 3, 13]. We tested the hypothesis that HGprt deficiency affects the development of the midbrain dopamine system in vivo, in a genetic HGprt-deficient mouse model for LND. The lack of HGprt disrupts multiple developmental events in the ventral midbrain, predominantly affecting proliferation and migration of presumptive mDA neurons. Subsequently, dopamine midbrain subregions appear abnormally specified and cortical dopaminergic innervation is affected. We hypothesize that these developmental structural abnormalities due to HGprt deficiency contribute to the neurobehavioral LND phenotype. A schematic summary of the main findings of the current study is depicted in Fig. 8.

### HGprt deficiency affects early proliferation and migration patterns of the midbrain DA system

Precise proliferation and migration patterns during development are fundamental in establishing the final complex organization of the dopaminergic ventral midbrain [23, 25]. Variations in these patterns may ultimately lead to an altered organization and functionality of the DA system. In the ventral midbrain of HGprt deficient embryos between E12.5 and E14.5, we found an increase in proliferation, both close to the VZ where the neural DA progenitors are generated and commence their migration, as well as in the presumptive VTA (Fig. 8) [30]. In addition, there was an increase in number of cells that had not exited the cell cycle, a step that is required to allow further differentiation [60]. This increase in proliferation was accompanied by an altered distribution and orientation of the dividing cells that suggested a premature deviation from their destined migratory route, resulting in a lower number of TH^+^ neurons in the area of the migratory path itself. Later in development, at E18.5, the increased proliferation in HGprt-deficient midbrains was no longer visible in most areas. Nevertheless, the TH^+^ cells occupied a larger region, again compatible with an aberrant migration pattern.

In order to explain the abnormal proliferation and migration in HGprt-deficient midbrains, we focused on the structural appearance of RG-L cells, as these cells are crucial for both [27]. RG-L cells provide neuronal progenitors close to the VZ, i.e., where the abnormal proliferation was most pronounced in HGprt-deficient midbrains and a scaffold to assist newborn mDA progenitors in their migration. A disruption of this RG-L scaffold has been reported to affect mDA neuronal migration before [28, 29, 61]. Indeed, our data revealed an abnormal RG-L scaffold in the midline of HGprt-deficient embryonic midbrains, with an increase in RG-L processes in caudal regions and a decrease in rostral regions.

Moreover, we observed an altered appearance and organization of β-catenin^+^ structures in the medial VZ. For the morphological integrity of the RG-L scaffold, β-catenin is an essential component of the cadherin-dependent cell adhesion complex involved in the attachment of the RG-L cell apical end-feet via adhere junctions within the VZ [28, 46]. Therefore, based on these data, we hypothesize that HGprt deficiency may affect RG-L function, resulting in the aberrant proliferation and migration patterns of DA progenitors shown here. Of note, β-catenin not only contributes to cell adhesion but is also a transcriptional regulator in the canonical Wnt pathway that regulates dopaminergic differentiation, particularly neuronal fate specification along the rostro-caudal axis [62]. In fact, it has been shown that an excess of β-catenin promotes neurogenesis, while the number of TH^+^ neurons are decreased due to interference with the expression of TFs that drive the dopaminergic development—a pattern that is similar to our findings [63]. It is tempting to speculate that dysregulation of the canonical Wnt pathway, as has been reported before in HGprt-deficient cell lines [18], is the primary defect due to HGprt deficiency.

Despite the clear abnormalities due to HGprt deficiency during embryonic midbrain development shown here, further study is required for a full comprehension of the nature of events and to distinguish whether there is merely a delay in development due to delayed cell cycle exit, a change in migrational patterns due to abnormal RG-L scaffolding, an inability to develop a proper neural dopaminergic programming due to abnormal canonical Wnt-driven differentiation, or a combination of these.

### HGprt deficiency causes neuroanatomical abnormalities later in midbrain development as well as in cortical target areas

In addition to aberrant early proliferation and migration patterns of mDA progenitors, HGprt deficiency also resulted in dopaminergic abnormalities later in development (Fig. 8). First, HGprt deficiency resulted in an altered structural organization of the developing dopaminergic subregions SN and VTA. The number of Sox6^+^ cells, marking the presumptive SN region, was increased while cells positive for VTA-specific Otx2^+^ showed decreased cell numbers, particularly towards the caudal extent. Moreover, these cells were abnormally distributed within the TH^+^ areas. Second, region-specific abnormalities in TH^+^ innervation of cortical areas were noted. In the mPFC, the organization of the TH^+^ fibers within the cortical wall was disrupted, while in the M1 and S1, there was a reduction in total TH^+^ fiber length. These data suggest that the early abnormalities in proliferation and migration are not only a temporary disturbance but illustrate that HGprt deficiency is also associated with neuroanatomical consequences later in time and in distant brain areas.

### Possible molecular mechanisms underlying abnormal brain developmental due to HGprt deficiency

The exact molecular mechanisms by which HGprt deficiency would cause the demonstrated neurodevelopmental defects need further study. In the absence of HGprt, hypoxanthine and guanine are degraded to uric acid rather than converted to their respective nucleotide pools and together with an activated de novo purine synthesis this leads to uric acid overproduction. It has been suggested that purine nucleotide depletion, or accumulation of toxic compounds could be responsible for brain dysfunction in LND [13]. However, this could not be confirmed in multiple patient-derived HGprt-deficient cell types in culture [64] or in HGprt-deficient knockout mice [65] for a long time. Only recently it has been reported that fibroblasts from LND patients do show ATP depletion and accumulation of 5-aminoimidazole-4-carboxamide riboside 5′-monophosphate (ZMP, an intermediary of the de novo purine biosynthetic pathway) when the culture medium has physiological levels of folic acid instead of typically artificially high levels as commonly used in cell studies [66]. The presence of 5-aminoimidazole-4-carboxamide ribonucleotide (AICAr, a ZMP derivative) in urine and cerebrospinal fluid of LND patients, but not in control individuals [66], as well as an increased AICAr content of HGprt deficient mouse brains compared to controls [67] suggest that similar metabolic aberrations may occur in both humans and mice in vivo.

These new findings allow further exploration of the molecular mechanisms by which HGprt deficiency causes abnormal brain development. For example, generic factors such as energy failure due to ATP depletion or toxicity of Z-nucleotides affecting mitochondrial activity may play a role. Moreover, Z-nucleotides may also affect the function of AMP-activated protein kinase (AMPK), a master regulator of cell metabolism involved in neuronal polarization and axonal growth [68]. A more specific mechanism could be that increased AICAr alters sonic hedgehog (SHH) and Wnt/β-catenin pathway gene expression, as has been shown in human embryonic carcinoma cell line NT2/D1 during neural differentiation [69], thereby providing a mechanistic connection between the purinergic derangement due to HGprt dysfunction, the irregularities in RG-L morphology and subsequent abnormal proliferation and migration of DA progenitors as discussed above. Additional studies are required to establish the exact HGprt-dependent neurodevelopmental mechanism that causes brain dysfunction in LND, as well as to investigate the possible therapeutic effect of folic acid to correct the purinergic abnormalities observed in HGprt-deficient cell models [66].

### Relevance of the current findings for the clinical phenotype of LND patients and future treatment opportunities

The current study is the first in providing direct in vivo evidence that HGprt deficiency is associated with an abnormal early development of the brain’s mDA system, a conceivable explanation of the selective DA deficiency in LND that has been known for a long time [13]. Both the extent as well as the timing of these events appear important determinants of the clinical phenotype and future treatment strategies, for several reasons.

First, abnormal dopaminergic innervation will affect the function of basal ganglia circuitry and it has been advocated that the LND clinical phenotype is due to profound dysfunction of these pathways, that serve motor, cognitive and behavioral aspects of behavior [13]. Specifically, the movement disorder dominated by dystonia in LND has been associated with dysfunction of basal ganglia *motor circuits*, connecting motor and somatosensory cortices with the putamen, while the attentional and executive impairments as well as the aberrant and self-injurious behaviors in LND have been attributed to dysfunction of *cognitive and behavioral circuits*, encompassing the PFC, caudate and ventral striatum. This hypothesis is fully supported by the abnormalities demonstrated here, where dopaminergic midbrain abnormalities are accompanied by abnormal innervation of the cortical areas mentioned. It should be noted that, although we have focused on cortical dopaminergic target areas, other dopaminergic target areas such as the striatum deserve attention in future studies to further unravel the functional anatomy of LND. In addition, the lateral habenula may be relevant for LND, as this abundantly dopaminergic innervated hub connects with both basal ganglia and limbic system [70], that may lead to enhanced impulsive behavior or even self-injurious behavior when disrupted [71].

Second, the timing of the pathogenic events demonstrated here appear important for the LND phenotype. It has been known for a long time that DA deficiency at different ages results in distinctive movement disorders: DA depletion in adult animals or humans causes parkinsonism, while dopamine depletion in juvenile animals or children causes dystonia similar to LND [72], or even self-injurious behavior—the behavioral hallmark of LND [73]. These phenomena have been interpreted as neuroplastic processes—referring to receptor function, postreceptor signalling pathways, electrophysiological function and synaptic changes—in response to the DA depletion during early development [74]. Our findings might illustrate the first events that lead to these neuroplastic changes and will direct the development of future treatment strategies for LND. As for other neurodevelopmental disorders, there is likely a critical window of opportunity for restorative therapies, because abnormal brain development is difficult to reverse once established [75]. If the DA defect in LND derails the subsequent innervation and development of dopaminergic target areas, this could explain the current inability to treat LND with the DA precursor levodopa, usually administered after symptoms arise [74]. Future studies should therefore focus on the potential reversibility of the early aberrations of dopaminergic maldevelopment, as well as the timing of such intervention, e.g. by gene therapy to prevent the dopaminergic midbrain maldevelopment demonstrated here, by administering levodopa very early before symptoms emerge to prevent unfavorable neuroplastic processes, or perhaps by administering supplements such as folic acid that may prevent pathogenic purinergic metabolic derangements [66].

## Conclusion

In summary, we show that HGprt deficiency in mice disrupts proper proliferation and migration patterns of developing mDA neurons during embryogenesis, affecting later mDA subpopulation development and organization, as well as dopaminergic innervation of the cerebral cortex. In absence of HGprt the RG-L process scaffold supporting mDA migration was affected, with differences along the rostro-caudal axis. These in vivo data provide direct evidence for the neurodevelopmental nature of the brain disorder in LND—a notion that has profound implications for future treatment approaches. Future studies should not only focus the specific molecular mechanisms underlying the neurodevelopmental abnormalities but also on optimal timing of therapeutic interventions to rescue DA neuron defects, which may also be relevant for other neurodevelopmental disorders.

### Supplementary Information

Below is the link to the electronic supplementary material.

Supplementary file1 (PDF 1233 KB)

Supplementary file2 (PDF 115 KB)

Supplementary file3 (PDF 612 KB)

Supplementary file3 (DOCX 19 KB)

## Data Availability

The datasets generated during and/or analyzed during the current study are available from the corresponding author on reasonable request.
