# Supplementary figures and images for: HGprt deficiency disrupts dopaminergic circuit development in a genetic mouse model of Lesch–Nyhan disease
Source: Cell Mol Life Sci. 2022 Jun 4;79(6):341. doi: 10.1007/s00018-022-04326-x (PMC9167210; doi:10.1007/s00018-022-04326-x)

Figure S1

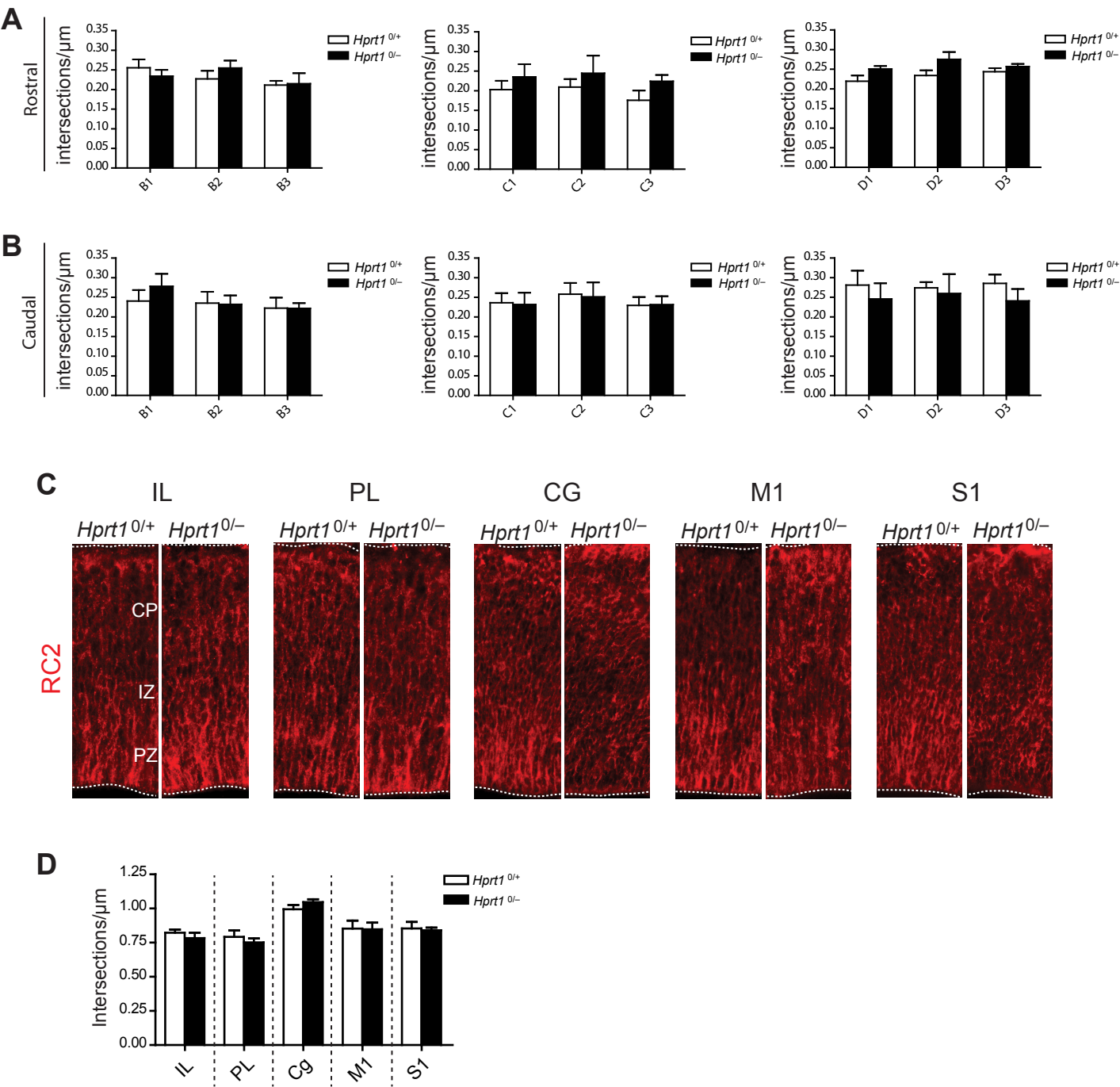

Supplement: Supplementary file 1 — Supplementary file1 (PDF 1233 KB) [file 18_2022_4326_MOESM1_ESM.pdf]

Figure S2

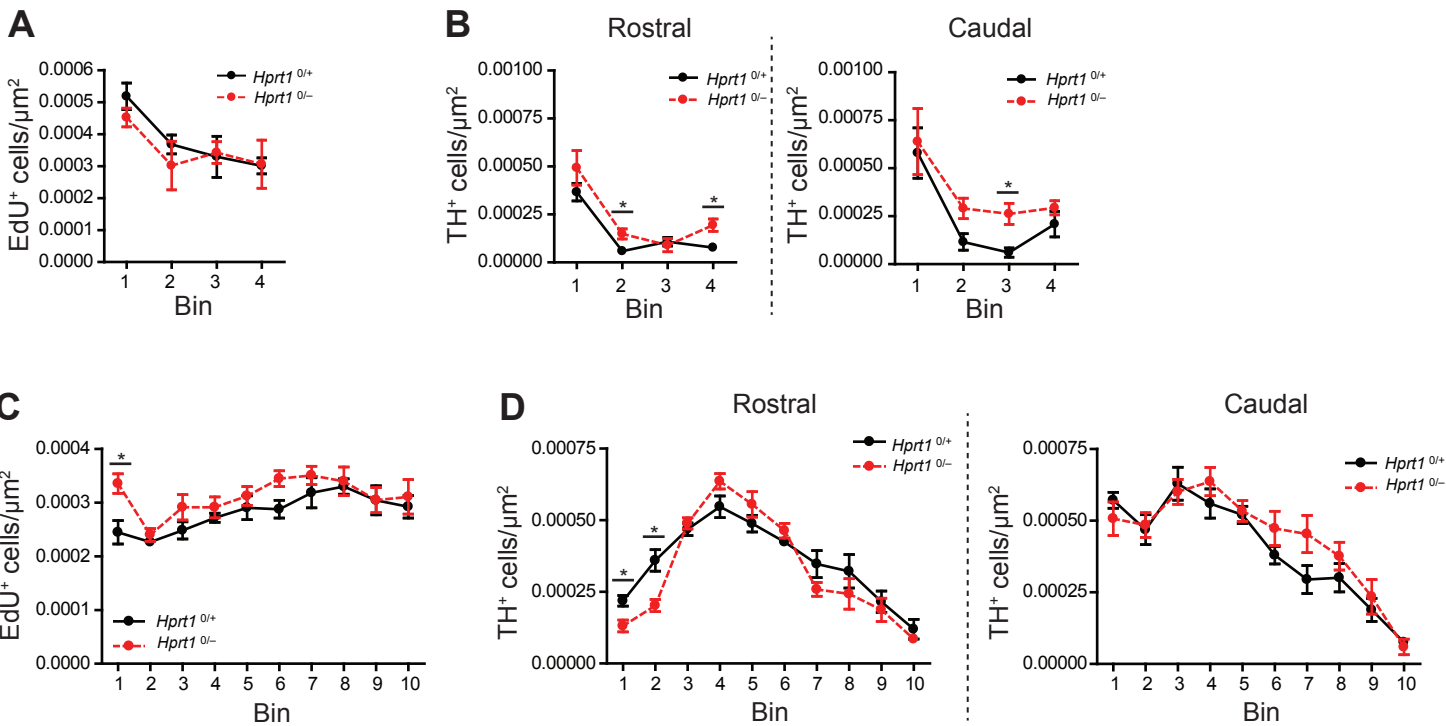

Supplement: Supplementary file 2 — Supplementary file2 (PDF 115 KB) [file 18_2022_4326_MOESM2_ESM.pdf]

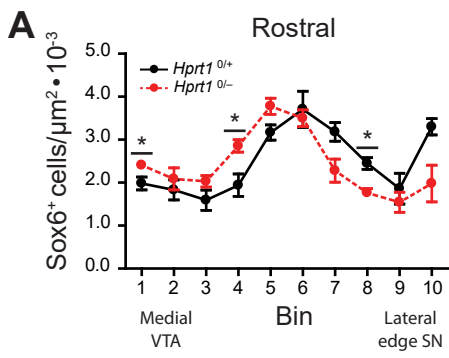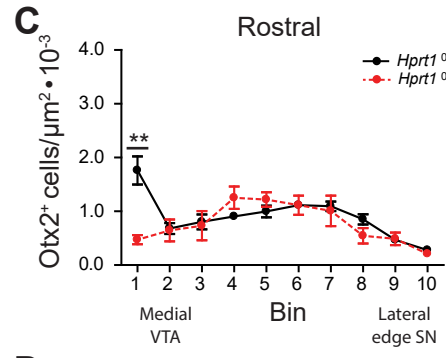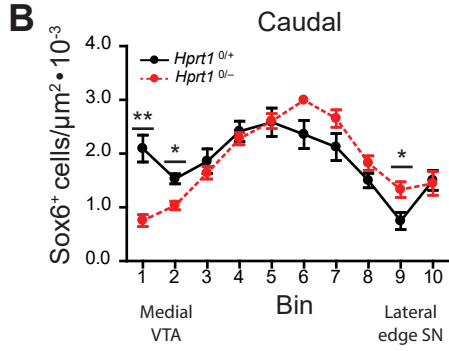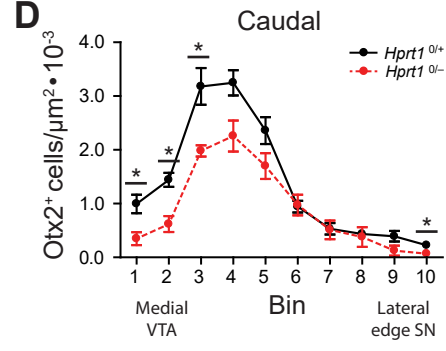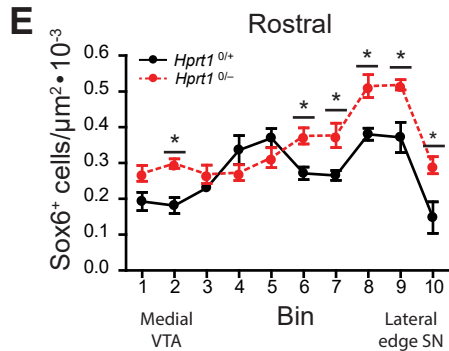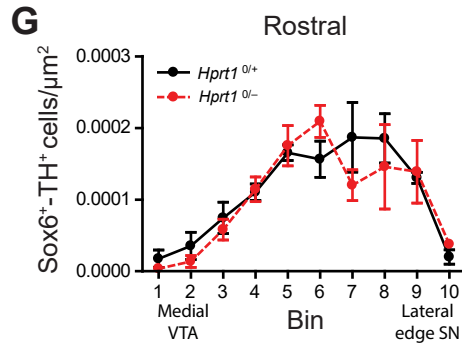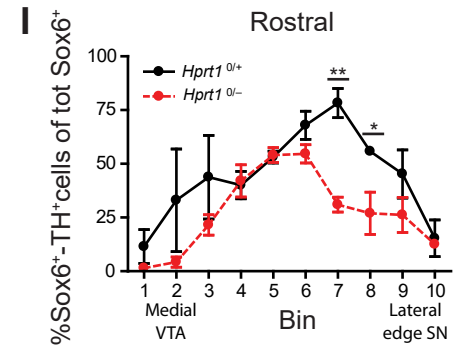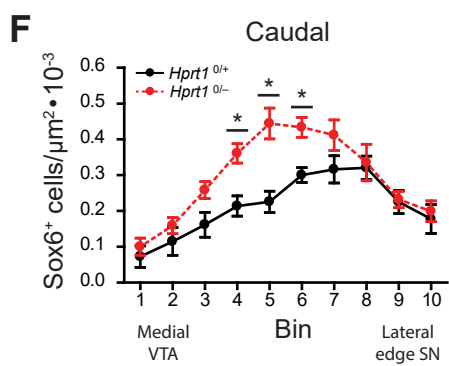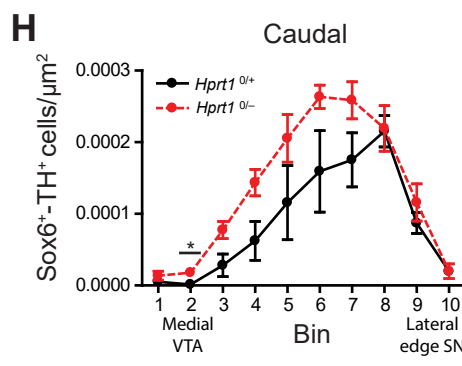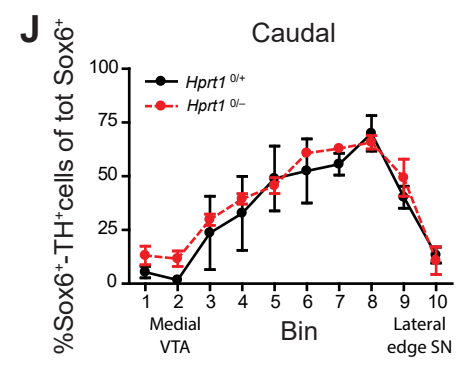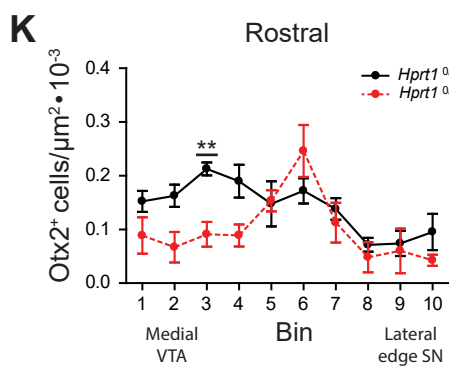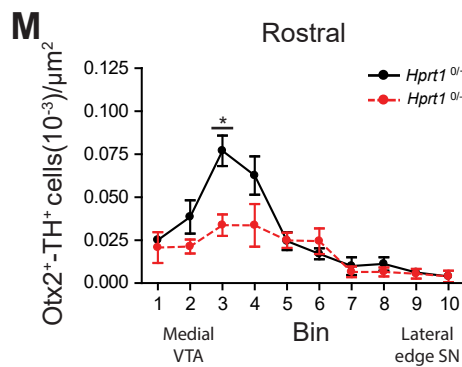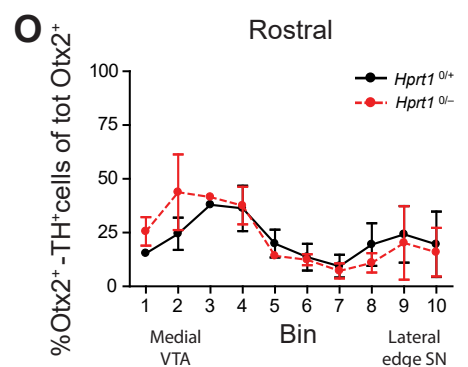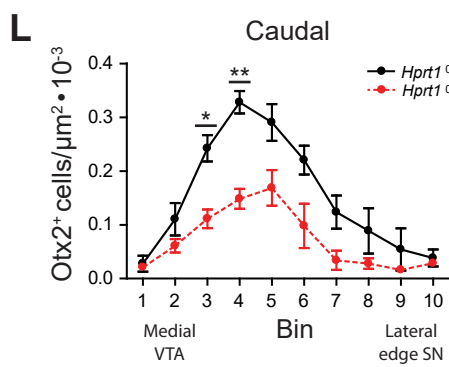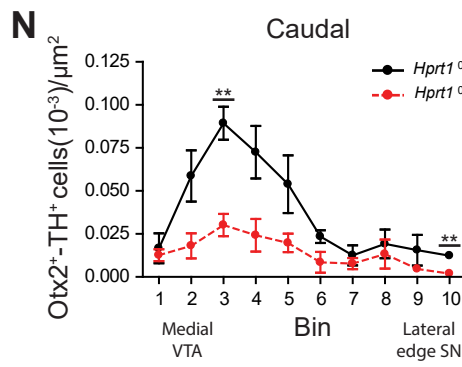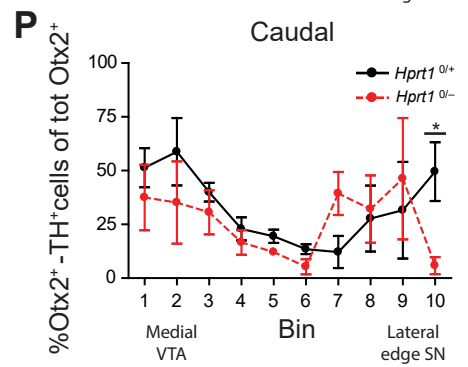

Supplement: Supplementary file 3 — Supplementary file3 (PDF 612 KB) [file 18_2022_4326_MOESM3_ESM.pdf]
