# Supplementary material for: HGprt deficiency disrupts dopaminergic circuit development in a genetic mouse model of Lesch–Nyhan disease
Source: Cell Mol Life Sci. 2022 Jun 4;79(6):341. doi: 10.1007/s00018-022-04326-x (PMC9167210; doi:10.1007/s00018-022-04326-x)
Supplement: Supplementary file 4 — Supplementary file3 (DOCX 19 KB) [file 18_2022_4326_MOESM4_ESM.docx]

**TABLES**

**Table 1**: PCR primer sequences for sex and HPRT1 genotyping.

| *Target* | *Sequence* |
| --- | --- |
| *Male Sry FW* | TGGGACTGGTGACAATTGTC |
| *Male Sry RV* | GAGTACAGGTGTGCAGCTCT |
| *Male/Female IL3 FW* | GGGACTCCAAGCTTCAATCA |
| *Male/Female IL3 RV* | TGGAGGAGGAAGAAAAGCAA |
| *MmHPRT1 (A2) FW* | TCATGGACTGATTATGGACAGG |
| *MmHPRT1-MUT-FW* | CAAATAAGATTTATTTATTATGAATACAGTATTTCTCC |
| *MmHPRT1-RV1* | GCTTATATTCAAACATCAACTACTCAACAG |
| *MmHPRT1-RV2* | CAACTACTCAACAGTTGCTAAGTTAGAATTAATAC |

**Table 2**: Primary antibodies, dilution and their supplier

| *Antibody* | *Dilution* | *Company* |
| --- | --- | --- |
| Rabbit-TH | 1:1000 | Pelfreeze |
| Chicken-TH | 1:500 | Abcam |
| Rabbit-RC2 | 1:50 | DSHB |
| Rabbit-Sox6 | 1:500 | Sigma |
| Rabbit-Otx2 | 1:400 | Sigma |
| Rabbit-Ki67 | 1:1000 | Abcam |
| Mouse-BrdU | 1:200 | NovusBio |
| Rat-Ctip2 | 1:500 | Abcam |
